# The impact of an e-newsletter or animated video to disseminate outdoor free-play information in relation to COVID-19 guidelines in New South Wales early childhood education and care services: a randomised controlled trial

**DOI:** 10.1186/s12889-023-16177-7

**Published:** 2023-07-07

**Authors:** Kathryn Reilly, Jacklyn Jackson, Melanie Lum, Nicole Pearson, Christophe Lecathelinais, Luke Wolfenden, Heidi Turon, Sze Lin Yoong

**Affiliations:** 1 School of Medicine and Public Health, College of Health, Medicine and Wellbeing, University of Newcastle, Callaghan, NSW Australia; 2 Priority Research Centre for Health Behaviour, University of Newcastle, Callaghan, NSW Australia; 3 Hunter Medical Research Institute (HMRI), New Lambton Heights, NSW Australia; 4 Hunter New England Population Health Unit, Hunter New England Local Health District, Wallsend, NSW Australia; 5 Global Obesity Centre, Institute for Health Transformation, School of Health and Social Development, Faculty of Health, Deakin University, Geelong, Australia

**Keywords:** Dissemination, Guideline Adoption, Randomised Controlled Trial, Early Childhood Education and Care, Physical activity, Outdoor Free Play

## Abstract

**Background:**

State-based Guidelines were issued for Early Childhood Education and Care (ECEC) services (caring for children 0–6 years) recommending greater time outdoors and inclusion of indoor-outdoor programs to facilitate social distancing to reduce spread of COVID-19. The aim of this 3-arm randomised controlled trial (RCT) was to examine the impact of different dissemination strategies on increasing ECEC service intentions to adopt recommendations from the Guidelines.

**Methods:**

This was a post-intervention only RCT. A sample of eligible ECEC services in New South Wales (n = 1026) were randomly allocated to one of three groups; (i) e-newsletter resource; (ii) animated video resource; or (iii) control (standard email). The intervention was designed to address key determinants of guideline adoption including awareness and knowledge. Following delivery of the intervention in September 2021, services were invited to participate in an online or telephone survey from October-December 2021. The primary trial outcome was the proportion of services intending to adopt the Guidelines, defined as intention to; (i) offer an indoor-outdoor program for the full day; or (ii) offer more outdoor play time. Secondary outcomes included awareness, reach, knowledge and implementation of the Guidelines. Barriers to Guideline implementation, cost of the dissemination strategies and analytic data to measure fidelity of intervention delivery were also captured.

**Results:**

Of the 154 services that provided post-intervention data, 58 received the e-newsletter (37.7%), 50 received the animated video (32.5%), and 46 received the control (29.9%). Services who received the animated video had nearly five times the odds (OR: 4.91 [1.03, 23.34] p = 0.046) than those in the control group, to report having intentions to adopt the Guidelines. There were no statistically significant differences in awareness or knowledge of the Guidelines between either intervention or control services. Development costs were greatest for the animated video. The extent to which the dissemination strategy was viewed in full, were similar for both the e-newsletter and animated video.

**Conclusion:**

This study found potential for the inclusion of interactive strategies to disseminate policy and guideline information within the ECEC setting, in the context of the need for rapid communication. Further research should explore the added benefits of embedding such strategies within a multi-strategy intervention.

**Trial registration:**

Retrospectively registered with the Australian New Zealand Clinical Trials Registry (ANZCTR) on the 23/02/2023 (ACTRN 12,623,000,198,628).

**Supplementary Information:**

The online version contains supplementary material available at 10.1186/s12889-023-16177-7.

## Background

Engaging children in adequate physical activity in early childhood is associated with many physical and psychosocial benefits [1–3]. Physical activity in children may include anything that gets their bodies moving, breathing faster and speeds up their heart rate, such as running, skipping or kicking a ball. Benefits of physical activity include reduced prevalence of overweight and obesity, better mental health and wellbeing, as well as a lower risk of developing chronic diseases later in life [4, 5]. As such, public health guidelines exist in multiple countries, recommending children of all ages engage in adequate amounts of physical activity [6–8]. However, only 54% of pre-schoolers (aged between 2 and 5 years) internationally meet the minimum physical activity recommendations (i.e. 60 min per day) [9]. Additionally, within the context of the world-wide COVID-19 pandemic, evidence suggests that child physical activity levels were further limited due to increased time spent indoors and time spent in social isolation as a way of reducing the spread of COVID-19 [10], suggesting that more specific guidance supporting child physical activity during this time was required.

The Early Childhood Education and Care (ECEC) setting has been identified as an important setting to support and promote child physical activity, as it provides access to a large proportion of young children aged 3–5 years old (approximately 90% world-wide) [11] for sustained and regular periods of time. Further, many ECEC services internationally, that is childcare centres that provide education and care to children under compulsory school age (0–6 years), are expected to implement a variety of policies and practices to support young children to meet the minimum physical activity guidelines whilst in care, through the provision of supportive staff, daily schedules, resources and infrastructure [8]. Examples of such recommended practices include providing more frequent opportunities for outdoor free play, given systematic reviews of observational studies have shown that outdoor time is positively associated with child physical activity [12]. European data also found that during periods of COVID-19 quarantine and self-isolation, children who followed a daily routine that factored in regular play periods of outdoor play for more than 2 h per day were more likely to meet physical activity recommendations [13].

One way of facilitating greater time outdoors to increase physical activity and reduce risk of infectious disease transmission in the ECEC setting may include introducing ‘indoor-outdoor’ (continuous/free-flow) free play routines, where children are able to move freely between the indoor and outdoor environments and choose where they wish to play, increasing the total time available for outdoor free play [2, 14]. Previous randomised trials have reported that implementing such routines may increase child levels of moderate-to-vigorous physical activity by approximately 5–6 min per day [2]. A number of key agencies have also recommended that ECEC services offer greater time outdoors and offer indoor-outdoor programs across the full day to help children spread out and facilitate social distancing to minimise the spread of COVID-19 in ECEC settings [15].

Dissemination has been defined as an “active approach of spreading evidence-based interventions or knowledge to the target audience via determined communication channels using planned strategies” [16]. In contrast to passive communication approaches which are non-targeted and generic in nature (e.g. standard mail out or distribution of information), dissemination strategies are designed to target provider and policy maker awareness, knowledge, attitudes, and intention to adopt policies and evidence based practices [17, 18]. The United States National Institute of Health defines dissemination as a distinct target behaviour from implementation, and a critical step to ensure the broader reach and adoption of guidelines [19]. As factors including guideline complexity, guideline organisation/evidence credibility and action-ability of / the guidelines tend to influence attitudes and knowledge of guidelines, dissemination strategies that target these factors have the potential to increase intentions to adopt recommendations in the ECEC setting [20, 21]. Despite this, there is an absence of evidence regarding the most effective dissemination strategies, with most studies relying on passive, non-targeted provision of information to communicate policy changes in ECEC settings [17, 22].

With the emergence of COVID-19 and rapidly changing health and social contexts, the importance of understanding how to quickly disseminate guideline recommendations to ECEC services in a resource-efficient and time-effective manner was identified, and provided an opportunity to assess the impact of strategies to disseminate health-related information to the sector.

As such, the aim of this 3-arm randomised controlled trial was to examine the use of different types of dissemination strategies: (i) an e-newsletter; and (ii) an animated video resource, compared with the provision of a standard email (passive diffusion), to assess the impact on increasing ECEC service intentions to adopt an indoor-outdoor program for the full day and offer more time outdoors. The e-newsletter and animated video formats were chosen as the dissemination resources as both are easily distributed via email, at scale, and are delivered via the same modality (email) as the control message. It was hypothesised that more targeted, engaging dissemination strategies (e.g. the e-newsletter or animated video resource) would increase ECEC service intentions to adopt the Guidelines relative to a passive standard email (control).

## Methods

The trial protocol was retrospectively registered on the Australian New Zealand Clinical Trials Registry (ANZCTR) on the 23/02/2023 (ACTRN 12,623,000,198,628 [23]. Ethics approval was provided by the Hunter New England Human Research Ethics Committee (2019/ETH12353) and the University of Newcastle (H-2008-0343).

### Study design and participants

This three-arm parallel group randomised controlled trial was conducted in September 2021, with post-intervention data collection only (October-December 2021).

In July 2021, updated COVID-19 specific Guidelines for New South Wales (NSW) ECEC services (hereafter referred to as the ‘Guidelines’) were released by the NSW Department of Education, and were disseminated by a number of ECEC based bodies. These Guidelines included a variety of recommendations (e.g. staff mask wearing indoors), as well as clear recommendations for ECEC services to adopt an indoor-outdoor program and include more time spent outdoors to help mitigate COVID-19 transmission in NSW ECEC services. This trial sought to evaluate whether two different dissemination strategies (a targeted e-newsletter or animated video) resulted in greater intentions to adopt the Guidelines (i.e. an indoor-outdoor program; and/or more time outdoors) in NSW ECEC services compared with a standard email.

A total of 1,400 ECEC services were randomly extracted from a publicly available National register (Australian Children’s Education & Care Quality Authority (ACECQA)) [24]. Services were removed from the sample if they were: (1) not listed as either a preschool or long day care service (e.g. family day care service, or before and after school care); (2) listed as a Department of Education service; (3) temporarily closed; (4) located outside of the state of NSW; or (5) were concurrently selected to participate in a nationwide survey being conducted by the research team. ECEC services located within the Hunter New England Local Health District of NSW were also excluded from the current randomised controlled trial, as they were already participating in a separate intervention to improve outdoor free play [25]. The remaining 1026 eligible ECEC services, caring for children 0–6 years, therefore make up the sampling framework for this study.

### Randomisation and allocation

Prior to services receiving the invitation to complete the follow-up survey, the sample of eligible ECEC services (n = 1026) were randomly allocated to one of three groups; (i) an e-newsletter resource; (ii) an animated video resource; or (iii) control (i.e. standard email containing the Guidelines as per the Department of Education website for indoor-outdoor programming and outdoor activities). Randomisation of services was stratified by area socio-economic status (SES) (classified using service postcode according to the Socio-Economic Indexes for Areas (SEIFA) categorisation [26]) as this has been shown to be associated with implementation of indoor-outdoor programs in Australian ECEC services [26, 27]. Randomisation was conducted by a blinded research statistician using a random number function in Microsoft excel in a 1:1:1 ratio.

### Recruitment

A total of 713 services (approximately 69.5% of eligible services) were emailed an invitation to participate in the follow-up survey within the three month follow-up period (i.e. before the 21st of December 2021).The email invitation included a link that directed participants to the information statement, and led them to complete the survey online. If service Nominated Supervisors had not completed the online survey approximately one week following the initial invitation email, services were called by trained interviewers inviting them to complete the survey via a Computer Assisted Telephone Interview. Nominated Supervisors are a sector specific role, responsible for the day-to-day management of a service and hence the preferred person to complete the survey.

### Intervention dissemination strategies

Intervention resource development was guided by the ‘Model for Dissemination of Research’ as outlined by Brownson et al. [28], and Leeman et al. ‘Interactive System Framework’ [18]. The ‘Model for Dissemination of Research’ defines the *source* (i.e. the agency, organisation or individual responsible for creating/distributing the new knowledge or product; e.g. NSW Health; NSW Department of Education), *message* (i.e. the information being disseminated; e.g. COVID-19 Guidelines for ECEC services), *audience* (i.e. the intended users; e.g. Nominated Supervisors in ECEC services) and the *channel* (i.e. the modality in which the message is communicated; e.g. e-newsletter or animated video) [29]. Additionally, the ‘Interactive System Framework’ recommends the use of two components as part of a dissemination strategy, including; (i) developing messages and materials customised to the target audience; and (ii) distribution of messages and materials through channels of optimum reach [18]. As such, the language and terminology used in both the e-newsletter and animated video was that which is common to the target audience (Nominated Supervisors) in the ECEC setting.

The intervention resources focused on addressing key barriers associated with Guideline adoption within the ECEC setting [29] (See Table 1), and were mapped to domains of the Theoretical Domains Framework (TDF). The TDF is a synthesis of 33 theories and 128 key theoretical constructs related to behaviour change, designed to assess implementation barriers and inform intervention design [30]. Both intervention resources addressed the same barriers to Guideline adoption, however the animated video resource, a novel approach to delivering guideline information, was developed to elicit an emotional reaction through the inclusion of audio and visual elements, including a visual demonstration of the behaviour [31]. For example, the animated video included scenes depicting an ECEC service, showing children happily and easily moving between the indoor and outdoor area and social distancing. To improve relatability to the Nominated Supervisors receiving the resource, the animated video also used the ECEC Educator first person voice.

**Table 1:** Development of the intervention resources

Barriers to Guideline adoption (30)	Content to address barriers	Resource: e-newsletter	Resource: animated video
Lack of awareness of the Guidelines (TDF Domain: Knowledge)	Make staff aware of the Guidelines, using language that ECEC educators and supervisors can relate to and understand.	Guidelines made explicit in text.	Guidelines stated in first person audio and in text via subtitles.
Lack of knowledge and understanding of the Guidelines (TDF Domain: Knowledge)	Promote the Guidelines, and the practices related to the Guidelines, using language that ECEC educators and supervisors can relate to and understand. Increase salience of the Guidelines and how they apply to ECEC services.	Guidelines made explicit in text. Explanation of what an indoor-outdoor program consist of.	First person narration from an educator. Images of educators implementing indoor-outdoor play (demonstration of behaviour) e.g. open doors from the indoors to outdoors with happy children outside.
Lack of understanding regarding the benefits of adopting the Guidelines (TDF Domain: Beliefs about consequences)	Promote the benefits to adopting the Guidelines (e.g. benefit to child wellbeing and health; facilitate social distancing).	Listed benefits of outdoor play and time outdoors for children.	Additional dialogue on potential stress on services related to COVID-19, no better time to increase opportunities for children to access the outdoors, and benefit to educators to look after themselves during this time and get their bodies moving. Positive images of children spread out outdoors, being active and happy.
Lack of belief they are capable for adopting the Guidelines (TDF Domain: Beliefs about capabilities)	Include simple tips for adopting the Guidelines. Use language that is strength-based and action focused, providing simple examples to facilitate adoption.	Simple examples given to help services get started with an indoor-outdoor program or more time outdoors.	Images of swapping scheduled indoor time with indoor-outdoor time, images of educators discussing what is best for their service (demonstration of behaviour).
Adopting the Guidelines is not considered to be a priority by service management, staff and attending families. (TDF Domain: Social Influences)	Highlight the source of the Guideline, the benefits of adopting the Guideline in relation to the present context i.e. COVID-19 risk of transmission	Reiterate the source of the Guidelines and the benefits in relation to COVID-19.	Additional dialogue on potential stress on services related to COVID-19, and no better time to increase opportunities for children to access the outdoors. Positive images of children and educators getting enjoyment from moving and being outside with nature used throughout the animated video.

All 1026 ECEC services received an email containing a link to the Guidelines. For both intervention groups, an additional link to either the e-newsletter or animated video was embedded within the email. All emails were sent to services on the 21st September 2021.

### Control group

ECEC services allocated to the control group received a generic email outlining the published Guidelines and a link to the NSW Department of Education website. While some constructs including awareness and knowledge may have also been targeted in this email, evidence suggests that generic emails of this nature typically are insufficient to influence intentions [17]. Control message can be viewed in Additional file 1.

### Data collection and measures

Outcomes were assessed post-intervention only, between October and December 2021 (within 3 months following the distribution of the intervention dissemination strategies) as this was considered a period where the COVID-19 specific Guidelines were most relevant to change in ECEC service practices. Data was collected via a survey completed by service Nominated Supervisors or delegate, either online or via telephone, administered according to a standard protocol by trained interviewers who were blinded to group allocation.

### Outcome measures

Assessment of the trial outcomes of the intervention was informed by the RE-AIM evaluation model [32] and involved three of the RE-AIM domains: reach, adoption and implementation. As this intervention sought to increase the immediate reach of the Guidelines from a COVID-19 context to reduce transmission of the disease, the effectiveness and maintenance domains were considered not relevant to the trial. This was consistent with our pre-specified outcomes.

### Primary outcome

#### Intentions to adopt the Guidelines

The primary trial outcome was the proportion of services intending to adopt the Guidelines. Services were defined as intending to adopt the Guidelines if they reported having intentions to; (i) offer an indoor-outdoor program for the full day; or (ii) offer more outdoor play time [15], as services unable to offer an indoor-outdoor program may still plan on offering more outdoor play time during the ECEC day.

Specifically, Nominated Supervisors were asked: “*Which of the following statements best describes your intention to adopt an indoor-outdoor program for the full day at your service?*” and “*Which of the following statements best describes your intention to offer more time outdoors at your service?*” Similar to previous studies assessing intention to adopt a guideline or policy [33, 34], response options were categorised according to the five stages of behaviour change [35]: (1) Pre-contemplation: ‘I have not thought about adopting’; (2) Contemplation: ‘I am thinking about adopting’; (3) Preparation: ‘I am planning on adopting’; (4) Action: ‘‘I am currently adopting’; (5) Maintenance: ‘I have adopted for more than 6 months’ [31]. In line with previous studies [33, 34], responses were dichotomised into ‘adopters’ (services in the preparation or action stage of change) and ‘non-adopters’ (services in the pre-contemplation and contemplation stages). Services in the maintenance stage were excluded from the analysis, as they reported having adopted the Guideline for more than 6 months, which preceded the distribution of the dissemination strategy and release of the Guidelines.

### Secondary outcomes

#### Awareness and reach of the Guidelines

Nominated Supervisors were asked if they are aware of the Guideline to offer an indoor-outdoor program for the full day and more time outdoors as a recommended practice for facilitating social distancing within ECEC services.

Data analytics, including the total number of views, link clicks and video views were collected 2 weeks after the email was sent to services for both the e-newsletter (from Microsoft Office Sway) and animated video (from YouTube) to evaluate reach of the intervention messages.

#### Knowledge of the Guidelines

Nominated Supervisor knowledge of the Guidelines was assessed via a series of four statements, in which they were asked to identify which statement was most consistent with the Guidelines for ECEC services regarding offering an indoor-outdoor program for the full day and more time outdoors. The four statements included: (1) Consider operating an indoor-outdoor program during one free play session a day. Have as many activities as possible placed in the outdoor space; (2) Consider operating an indoor-outdoor program for the full day/session. Consider spending more time outdoors, consider the placement of activities and the amount of activities in the outdoor space (correct response); (3) Spend more time outdoors and plan activities designed to help keep children 1.5 m apart at all times; and (4) Masks must be worn when spending time outdoors, which will allow the children and staff to socialise freely.

Services that correctly identified the statement most consistent with the Guidelines (Statement 2.*)*, were assessed as having knowledge of the Guidelines.

#### Implementation of the Guidelines i.e. indoor-outdoor programs for the full day and/or offering all free play outdoors

In order to measure implementation of the Guidelines, that is; (i) offering an indoor-outdoor program for the full day; and (ii) offering more time outdoors, Nominated Supervisors were asked to provide an estimation of the total time (in minutes) provided for unstructured child-initiated free play per day in the past week. Following this, services were asked to report how much of this free play time (in minutes) was offered as indoor-only free play; outdoor-only free play; or indoor-outdoor free play.

Services were categorised as implementing the Guidelines if they reported all free play being offered as outdoor-only and/or indoor-outdoor for the full day. Services that reported providing any indoor-only free play time (> 0 min) were categorised as not implementing the Guidelines.

Survey items assessing Guideline implementation was assessed in a random sub-sample (approximately 50%) of services, in order to reduce survey length and burden to the Nominated Supervisor.

### Other outcomes

#### Barriers to implementing the Guidelines

The intervention resources were developed to target known barriers to the adoption of indoor-outdoor programs and offering more time outdoors in the ECEC setting. As per previous research conducted within the ECEC setting [36], we adapted previously validated survey items based on the TDF to explore service perceptions of barriers to implementing the Guidelines. Survey items assessed the following TDF domains: Beliefs about consequences (3 items); Beliefs about capabilities (3 items); and Social Influences (3 items) (See Additional file 2). Responses were scored on a 7-point Likert scale (1 = strongly disagree; 2 = disagree; 3 = slightly disagree; 4 = Neither agree nor disagree; 5 = slightly agree; 6 = Agree; 7 = strongly agree).

A mean score less than five was used to identify a domain as a barrier to Guideline implementation, that is, the Nominated Supervisor did not respond favourably to the item statement (responded neither agree nor disagree/slightly disagree/disagree/strongly disagree).

### Cost

In order to compare differences in cost of the dissemination strategies, intervention development and delivery costs were calculated for the 3 arms. The cost of intervention development and delivery was calculated by recording time spent by the research team to develop and distribute the email to services and cost of the software subscription to create the animated video.

### Fidelity

Two weeks after the intervention resource was emailed to services, data analytics including the average time spent viewing the dissemination strategies, and average completion (i.e. how far all views scrolled through the e-newsletter; and how long participants viewed the animated video) of the dissemination strategies were collected for both the e-newsletter (from Microsoft Office Sway) and animated video (from YouTube) to evaluate the fidelity of the intervention messages.

### Nominated Supervisor and service characteristics

Nominated Supervisors were asked to report the number of years they have been employed in their current role at the service and highest level of relevant qualification completed related to their role at the service. Service operation hours, the number of children and the ages of children that attend the service were also reported by the Nominated Supervisor.

Service postcodes were obtained from centralised records from ACECQA [24]. Service SES was based on service postcode. Similar to other Australian-based implementation studies [37, 38], ‘higher socio-economic status’ was classified as postcodes ranked in the top 50% of NSW, whilst ‘lower socio-economic’ status was classified as the bottom 50%, based on SEIFA developed by the Australian Bureau of Statistics [26]. Service postcode was also used to describe locality as either ‘rural’ (those that resided in outer regional, remote and very remote areas) or ‘urban’ (those that resided in inner regional or major cities) based upon the 2016 Accessibility/Remoteness Index of Australia [39].

### Sample size calculation

Approximately 500 services were expected to provide post-intervention data, to allow for approximately 165 services per arm (assuming that response rates are equally distributed across the three arms) included in the evaluation. For the primary outcome (the proportion of services adopting the Guidelines regarding indoor-outdoor play for the full day and more time outdoors), 165 childcare services per arm was calculated to detect difference in service Guideline adoption of 14.2%, based on previous data indicating that 62.3% of ECEC services had adopted an indoor-outdoor program [27], with a significance level of 5% and 80% power.

### Statistical analysis

Analysis was undertaken using SAS version 9.3 by a blinded statistician. Analyses were performed on services that completed the post-intervention follow-up survey, with ECEC services as the unit of analysis. Descriptive statistics were used to describe service demographics and Nominated Supervisor characteristics of the sample.

Intervention effects on the primary trial outcome were assessed using separate logistic regression analyses to examine differences in Guideline adoption (e-newsletter vs. animated video; e-newsletter vs. control; animated video vs. control), controlled for service size, location/remoteness, and date of survey completion.

Dichotomous secondary outcomes (awareness, knowledge and implementation of the Guideline) were assessed using separate logistic regression analyses similar to the primary outcome.

Continuous secondary outcomes (i.e. barriers to implementing the Guidelines) were calculated for each service by averaging the 3 items of each targeted TDF domain. Differences between the three arms were assessed through linear regression models, controlled for service size, location/remoteness, and date of survey completion.

## Results

**Fig. 1:**
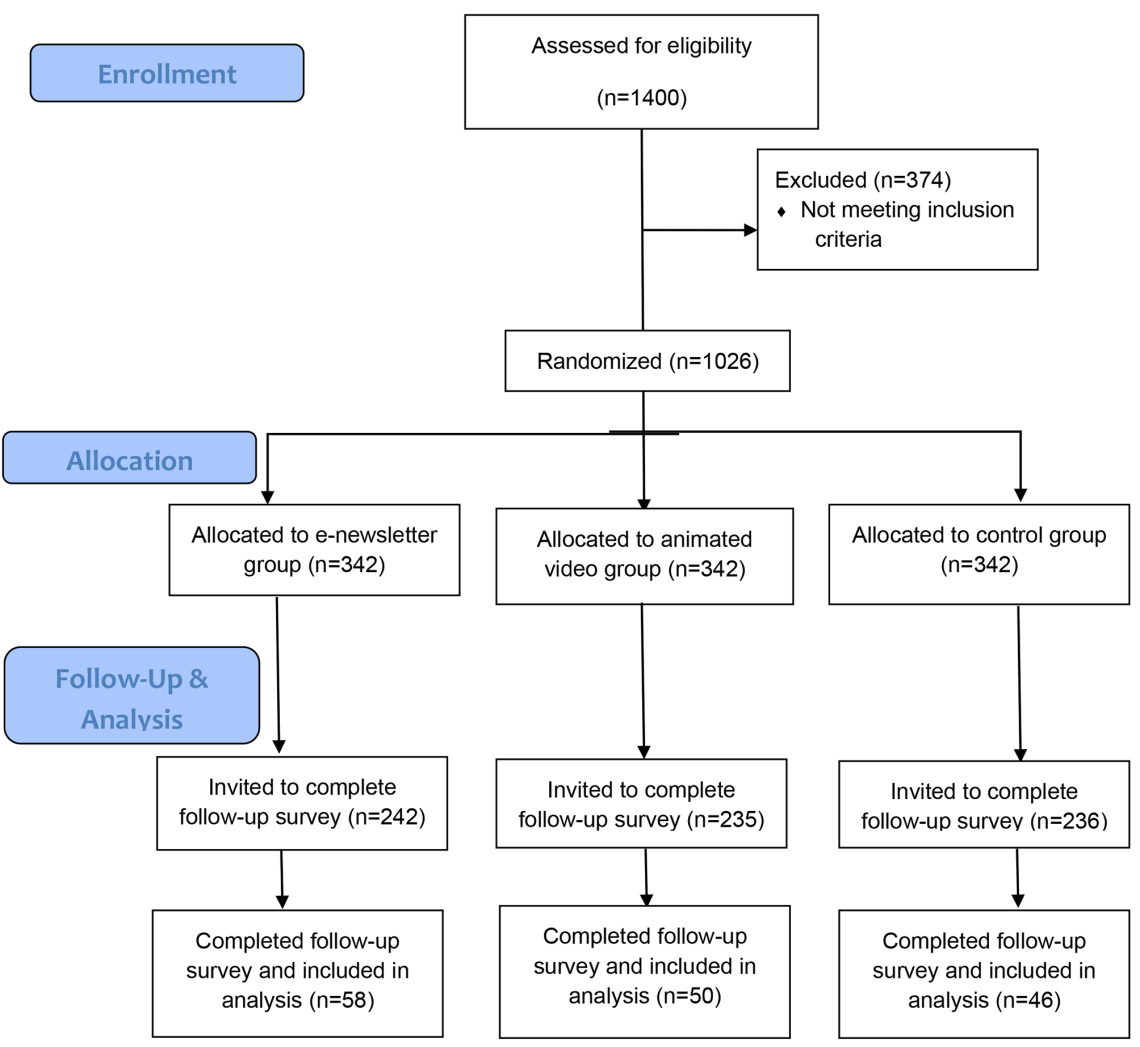
Flow chart of study participants

Of the 713 services invited to complete the follow-up survey, within 3 months of sending out the dissemination intervention (21st December 2021), a total of 154 services consented to and completed the follow-up survey (21.6%). Of these 154 participating services, 58 received the e-newsletter (37.7%), 50 received the animated video (32.5%), and 46 received the control (29.9%) (Fig. 1). Nominated Supervisor and service characteristics are presented in Table 2. There were no observed differences in service demographics between consenting and non-consenting services at follow-up.

**Table 2:** ECEC Service and Nominated Supervisor Characteristics

	e-newsletter group (n = 58)	Animated video group (n = 50)	Control group (n = 46)	All services (n = 154)
**Service Type**	N (%)	N (%)	N (%)	N (%)
Preschool	17 (29.31%)	12 (24.00%)	9 (19.57%)	38 (24.68%)
Long Day Care	41 (70.69%)	38 (76.00%)	37 (80.43%)	116 (75.32%)
**Service SES**				
Low SES	26 (44.83%)	21 (42.00%)	16 (34.78%)	63 (40.91%)
High SES	32 (55.17%)	29 (58.00%)	30 (65.22%)	91 (59.09%)
**Service locality**				
Urban	39 (67.24%)	39 (78.00%)	35 (76.09%)	113 (73.38%)
Rural	19 (32.76%)	11 (22.00%)	11 (23.91%)	41 (26.62%)
**Age of children attending the service**				
2 years and under	44 (75.86%)	39 (78.00%)	35 (76.09%)	118 (76.62%)
Over 2 years old	58 (100%)	50 (100%)	45 (97.83%)	153 (99.35%)
**Opening hours per day.**	**Mean (SD)**	**Mean (SD)**	**Mean (SD)**	**Mean (SD)**
	10.02 (1.54)	9.79 (1.98)	9.93 (1.53)	9.92 (1.69)
**Average number of children/day**	49.52 (22.28)	49.40 (23.00)	42.35 (17.71)	47.34 (21.38)

### Primary trial outcomes: intention to adopt the guidelines

As shown in Table 3, services who received the animated video had nearly five times the odds (OR: 4.91 [1.03, 23.34] p = 0.046) than those in the control group, to report being in the preparation or action phase of adopting the practice of offering more time outdoors. Services in the e-newsletter group also reported greater levels of adoption for offering more time outdoors than the control group (OR: 3.49 [0.86, 14.09] p = 0.08), but this was not statistically significant. There was no statistically significant difference in service intentions to adopt offering an indoor-outdoor program for the full day between the three groups.

**Table 3:** Primary outcome: Service intention to adopt the guidelines

Guideline adoption N (%)	e-newsletter group (n = 58)	Animated video group (n = 50)	Control group (n = 46)	Between Group Estimate (OR): e-newsletter vs. control	Between Group Estimate (OR): Animated video vs. control	Between Group Estimate (OR): animated video vs. e-newsletter
Offering an indoor-outdoor program for the full day	20 (54.05%)	17 (48.57%)	14 (50%)	1.12 (0.40, 3.14); p = 0.83	0.79 (0.28, 2.26); p = 0.66	0.71 (0.27, 1.89); p = 0.49
Offering more time outdoors	24 (85.71%)	23 (88.46%)	15 (62.50%)	3.49 (0.86, 14.09); p = 0.08	4.91 (1.03, 23.34); p = 0.046	1.41 (0.27, 7.46); p = 0.68

Adoption: defined as services in preparation and action (3 and 4), non-adopters are pre-contemplation or contemplation (1 and 2). Maintenance or unable to offer (5 and 6 excluded)

#### Secondary trial outcomes: awareness, knowledge and implementation of the Guidelines

There were no statistically significant differences in awareness and knowledge of the Guidelines between either intervention group and control services (see Table 4).

**Table 4:** Secondary outcomes: Awareness, knowledge and implementation of the Guidelines

N (%)	e-newsletter group (n = 58)	Animated video group (n = 50)	Control group (n = 46)	Between Group Estimate (OR): e-newsletter vs. control	Between Group Estimate (OR): Animated video vs. control	Between Group Estimate (OR): Animated video vs. e-newsletter
Services aware of the Guidelines	53 (91.38%)	49 (98.00%)	40 (88.89%)	1.37 (0.34, 5.51); p = 0.66	6.19 (0.66, 58.16); p = 0.11	4.52 (0.48, 42.70); p = 0.19
Services with knowledge of the Guidelines	42 (72.41%)	32 (64.00%)	29 (64.44%)	1.45 (0.60, 3.52); p = 0.40	1.03 (0.43, 2.48); p = 0.94	0.71 (0.30, 1.67); p = 0.43
Proportion of services implementing as per the Guidelines	4/21 (19.05%)	7/27 (25.93%)	3/22 (13.64%)	1.68 (0.29, 9.66); p = 0.55	2.12 (0.44, 10.25); p = 0.35	1.26 (0.27, 5.78); p = 0.76

Whilst not statistically significant, more services that received the animated video implemented the Guidelines (i.e. i) offered an indoor-outdoor program for the full day; or ii) offered more time outdoors (26%, compared to 19% for the e-newsletter group and 14% for the control group) (see Table 4).

*Other trial outcomes: Barriers to implementation, cost of intervention, reach and fidelity.*

There were no statistically significant differences between groups in the reporting of common barriers to adoption of indoor-outdoor programs and offering more time outdoors. However, for the control group, for two of the domains measured; ‘Beliefs about capabilities’ and ‘Social influences’, the mean score was below 5, indicating they are a barrier to Guideline implementation (see Table 5).

**Table 5:** Barriers to guideline implementation

Mean Score (SD)	e-newsletter group (n = 58)	Animated video group (n = 50)	Control group (n = 46)	Between Group Estimate (OR): e-newsletter vs. control	Between Group Estimate (OR): Animated video vs. control	Between Group Estimate (OR): Animated video vs. e-newsletter
Beliefs about consequence	5.82 (1.17)	5.68 (1.09)	5.74 (1.23)	0.03 (-0.44, 0.50); p = 0.90	-0.10 (-0.58, 0.38); p = 0.69	-0.13 (-0.58, 0.33); p = 0.58
Beliefs about capabilities	5.40 (1.27)	5.21 (1.48)	4.96 (1.62)	0.35 (-0.23, 0.93); p = 0.23	0.17 (-0.43, 0.76); p = 0.58	-0.18 (-0.74, 0.37); p = 0.51
Social influences	5.39 (1.30)	5.28 (1.41)	4.98 (1.48)	0.31 (-0.25, 0.87); p = 0.27	0.20 (-0.38, 0.78); p = 0.49	-0.11 (-0.65, 0.43); p = 0.68

Based largely on research team salary and time, the development costs of the intervention arms were as follows; control email AUD$58.88, e-newsletter AUD$1,387.60 and animated video AUD$3,787.82. The higher cost to develop the animated video is due to staff time and the initial software purchase cost. The cost to deliver the emails was AUD$294.40 split across the three groups.

As can be seen in Table 6 below, total views and the extent to which the dissemination strategy was seen in full, were similar for both the e-newsletter and animated video.

**Table 6:** Reach and fidelity of the dissemination strategies

	E-newsletter group (approximate reading time = 3 min)	Animated video group (length = 3 min and 28 s)
Total unique views (reach)	183	139
Average view duration (fidelity)	2 min	2 min 17 s
Average ‘completion’ of intervention, i.e. % of e-newsletter read/ % of animated video watched (fidelity)	65%	66%
Proportion of viewers that read in-depth or watched until the end (fidelity)	51%	47%

Unique views for e-newsletter, counts as views from unique device. Unique views for animated video counts as views for 30 s or more. Average time viewers spent watching/viewing strategies. Average completion for e-newsletter, is how far all viewers scrolled through the e-newsletter. Average completion of the animated video, is the percent of each video the average viewer watched. In-depth read of e-newsletter, counted as the viewers that spent a significant time reading through and interacting with the content

## Discussion

This is the first study to assess the impact of targeted dissemination strategies (animated video or e-newsletter) on increasing ECEC service intentions to adopt an indoor-outdoor program for the full day and offer more time outdoors, compared with a standard email control in response to COVID-19. The study found the animated video group had a significantly higher intention to adopt more time outdoors than the control group, and had twice the odds of implementing indoor-outdoor programs, compared to control. While intentions to implement an indoor-outdoor program in the intervention groups was not statistically significantly different compared with control, these results appear to support the use of targeted dissemination strategies to improve ECEC service intentions for practice adoption that are important prerequisites for improving practice implementation [40].

These findings are consistent with a previous study assessing the impact of distribution of educational material on ECEC cook’s intentions to adopt nutritional guidelines [20]. Cooks in this study who received the educational material reported significantly higher intentions to use the guidelines, however no difference was found between groups in guideline implementation in relation to number of serves of fruit and vegetables on menus [20]. Combined with the findings from our current study, it appears that dissemination strategies (e.g. targeted animated video or e-newsletter) are promising in changing behavioural intentions. Additional implementation strategies may however be needed to support the conversion of intentions to adopt into practice implementation in ECEC services in order to address additional barriers to implementation. For example, environmental context and resource barriers related to service staffing, and service layout not being conducive to outdoor play, are known barriers to implementation experienced by services [25], and were not explicitly addressed within our tested dissemination strategies. Additionally, a recently published Cochrane review exploring the effectiveness of printed educational materials (PEMs) on the practice of healthcare professionals, indicated that while PEMs distributed to healthcare professionals likely improved practice, PEMs in computerised versions made little to no difference to practice compared to PEM in printed versions [21]. It appears unclear whether computer-based dissemination strategies, including those tested in the current trial, are more effective than traditional PEMS.

Surprisingly, this study found no differences between groups in knowledge or awareness of the Guidelines which were the key targeted constructs of the dissemination strategies. This may be due to multiple sources (e.g. NSW Department of Education, ECEC-based bodies) circulating the Guidelines to services simultaneously, serving as a source of trial contamination and addressed within the control group (as minimum care) [41]. Additionally intervention fidelity may have also impacted results. For both intervention dissemination strategies, it appears approximately two minutes was the cut-off for service staff engagement (average view duration for e-newsletter 2 min, for animated video 2 min 17 s (approx. 66% of the video)). As such, some information related to the Guideline may have been missed. This timeframe of 2–3 min is consistent with those recommended for digital storytelling [42], which has been tested as a novel and engaging educational tool to enhance knowledge and skills [43]. This evidence highlights that future dissemination interventions using interactive modalities (such as those used in the current trial) should prioritise the most important content to be delivered first and that message length be kept to under two minutes to support improved engagement with materials.

Whilst the cost of the animated video appears significantly higher than the e-newsletter, a large proportion of the cost was for the purchase of the software subscription (AUD$1012.00), which could be shared across all communication approaches. Additionally, it should be noted that once the animated video is developed, it can easily and at a negligible cost, be disseminated to a large number of recipients via email or other means. Further research is needed to identify the true value and public health benefit of such an investment within a larger sample of ECEC services.

Strengths of the study include its randomised controlled design and theory-informed intervention development and evaluation. Limitations of this study include the variation in time (1–3 months) between receiving the dissemination strategy and when Nominated Supervisors undertook the-post intervention survey. This may have impacted on study findings as services had variable time to implement changes to their free play schedules, however date of survey completion was controlled for in our analyses. We were also unable to reach our pre-specified sample size (n = 500) due to challenges with obtaining consent to participate in the survey in the specific three month time frame, due to factors such as COVID-19 related staff shortages, ECEC closures during lock downs and potentially staff fatigue and competing priorities during this time. As such our findings are underpowered and may be biased by those most likely to be engaged with the content.

In the context of COVID-19 it should also be noted that this was a time of regular communication regarding updates to restrictions and social distancing and hence, it is difficult to know if a different result may occur without the presence of a pandemic or if disseminating guidelines unrelated to COVID-19. Additionally, this was a time of transition and adaptation in terms of lock downs and tight restrictions related to COVID-19 within Australia, potentially resulting in ECEC staff shortages and interruptions, changes to policies and practices, all of which likely impacted on adoption and implementation of the Guidelines.

## Conclusion

This study found potential for the inclusion of interactive strategies such as e-newsletters and animated videos to disseminate policy and guideline information within the ECEC setting to increase intention to adopt outdoor free play policies. Further research should explore the added benefits of embedding such strategies within a multi-strategy implementation intervention as initial adoption is a pre-requisite to ongoing implementation. Findings from this study will inform future dissemination strategies to maximise the reach and adoption of health-related information within the ECEC setting, particularly where rapid communication is needed.

## Electronic supplementary material

Below is the link to the electronic supplementary material.

Supplementary Material 1

Supplementary Material 2

## Data Availability

Study materials and data will be held by the Administering Institution. De-identified data sets and all study material may be available on reasonable request from the corresponding author.

## List of abbreviations

ACECQA: Australian Children’s Education & Care Quality Authority
ECEC: Early Childhood Education and Care
NSW: New South Wales
PEMs: Printed Educational Materials
SEIFA: Socio-Economic Indexes for Areas
SES: Socio-Economic Status
TDF: Theoretical Domains Framework

## References

[1] Lindsay AC, Greaney ML, Wallington SF, Mesa T, Salas CF (2017). A review of early influences on physical activity and sedentary behaviors of preschool-age children in high‐income countries. J Spec Pediatr Nurs.

[2] Razak LA, Yoong SL, Wiggers J, Morgan PJ, Jones J, Finch M (2018). Impact of scheduling multiple outdoor free-play periods in childcare on child moderate-to-vigorous physical activity: a cluster randomised trial. Int J Behav Nutr Phys Activity.

[3] Jones J, Wyse R, Finch M, Lecathelinais C, Wiggers J, Marshall J (2015). Effectiveness of an intervention to facilitate the implementation of healthy eating and physical activity policies and practices in childcare services: a randomised controlled trial. Implement Sci.

[4] Anderson E, Durstine JL (2019). Physical activity, exercise, and chronic diseases: a brief review. Sports Med Health Sci.

[5] Sando OJ, Sandseter EBH (2020). Affordances for physical activity and well-being in the ECEC outdoor environment. J Environ Psychol.

[6] Tremblay MS, Chaput J-P, Adamo KB, Aubert S, Barnes JD, Choquette L (2017). Canadian 24-hour movement guidelines for the early years (0–4 years): an integration of physical activity, sedentary behaviour, and sleep. BMC Public Health.

[7] The Department of Health. Australian 24-Hour Movement Guidelines for the Early Years (Birth to 5 years): Australian Government. ; 2019 [Available from: https://www1.health.gov.au/internet/main/publishing.nsf/Content/health-pubhlth-strateg-phys-act-guidelines#npa05.

[8] Jackson JK, Jones J, Nguyen H, Davies I, Lum M, Grady A (2021). Obesity Prevention within the early Childhood Education and Care setting: a systematic review of Dietary Behavior and Physical Activity Policies and Guidelines in High Income Countries. Int J Environ Res Public Health.

[9] Tucker P (2008). The physical activity levels of preschool-aged children: a systematic review. Early Child Res Q.

[10] Bates LC, Zieff G, Stanford K, Moore JB, Kerr ZY, Hanson ED (2020). COVID-19 impact on behaviors across the 24-hour day in children and adolescents: physical activity, sedentary behavior, and sleep. Children.

[11] OECD- Social Policy Division - Directorate of Employment Labour and Soical Affairs. PF3.2: enrolement in childcare and pre-school. OECD Family Database; 2019.

[12] Gray C, Gibbons R, Larouche R, Sandseter EBH, Bienenstock A, Brussoni M (2015). What is the relationship between outdoor time and physical activity, sedentary behaviour, and physical fitness in children? A systematic review. Int J Environ Res Public Health.

[13] Kovacs VA, Starc G, Brandes M, Kaj M, Blagus R, Leskošek B (2022). Physical activity, screen time and the COVID-19 school closures in Europe–An observational study in 10 countries. Eur J sport Sci.

[14] Razak LA, Jones J, Clinton-McHarg T, Wolfenden L, Lecathelinais C, Morgan PJ (2020). Implementation of policies and practices to increase physical activity among children attending centre‐based childcare: a cross‐sectional study. Health Promotion Journal of Australia.

[15] NSW Government. COVID-19 guidelines for ECEC services 2021 [Available from: https://education.nsw.gov.au/early-childhood-education/coronavirus/advice-for-services-and-providers.

[16] Rabin BA, Glasgow RE, Kerner JF, Klump MP, Brownson RC (2010). Dissemination and implementation research on community-based cancer prevention: a systematic review. Am J Prev Med.

[17] Shelton RC, Lee M, Brotzman LE, Wolfenden L, Nathan N, Wainberg ML (2020). What is dissemination and implementation science?: an introduction and opportunities to advance behavioral medicine and public health globally. Int J Behav Med.

[18] Leeman J, Birken SA, Powell BJ, Rohweder C, Shea CM (2017). Beyond “implementation strategies”: classifying the full range of strategies used in implementation science and practice. Implement Sci.

[19] Department of Health and Human Services, National Institute of Health. Dissemination and implementation research in health. PAR-16-238 2016 [Available from: https://grants.nih.gov/grants/guide/pa-files/par-16-238.html.

[20] Yoong SL, Jones J, Marshall J, Wiggers J, Seward K, Finch M (2015). A theory-based evaluation of a dissemination intervention to improve childcare cooks’ intentions to implement nutritional guidelines on their menus. Implement Sci.

[21] Giguère A, Zomahoun HTV, Carmichael P-H, Uwizeye CB, Légaré F, Grimshaw JM et al. Printed educational materials: effects on professional practice and healthcare outcomes. Cochrane Database of Systematic Reviews. 2020(8). 10.1002/14651858.CD004398.pub4 PMC8475791 32748975

[22] Farewell CV, Puma J, Bergling E, Webb J, Quinlan J, Shah P (2020). An exploration of constructs related to dissemination and implementation of an early childhood systems-level intervention. Health Educ Res.

[23] Australian New Zealand Clinical Trials Registry (ANZCTR). Available from: ANZCTR.

[24] Australian Children’s Education and Care Quality Authority. National registers. In: National Quality Agenda, editor. Register data: New South Wales2021.

[25] Yoong SL, Pearson N, Reilly K, Wolfenden L, Jones J, Nathan N (2022). A randomised controlled trial of an implementation strategy delivered at scale to increase outdoor free play opportunities in early childhood education and care (ECEC) services: a study protocol for the get outside get active (GOGA) trial. BMC Public Health.

[26] Common wealth of Australia., Australian Bureau of Statistics. Socio-economic indexes for areas (SEIFA) 2016 [Available from: https://www.abs.gov.au/ausstats/abs@.nsf/mf/2033.0.55.001.

[27] Jones J, Wolfenden L, Grady A, Finch M, Bolsewicz K, Wedesweiler T (2020). Implementation of continuous free play schedules in australian childcare services: a cross-sectional study. Health Promotion Journal of Australia.

[28] Brownson RC, Eyler AA, Harris JK, Moore JB, Tabak RG (2018). Research full report: getting the word out: new approaches for disseminating public health science. J public health Manage Pract.

[29] Razak LA, Clinton-McHarg T, Jones J, Yoong SL, Grady A, Finch M (2019). Barriers to and facilitators of the implementation of Environmental Recommendations to encourage physical activity in Center-Based Childcare Services: a systematic review. J Phys Activity Health.

[30] Atkins L, Francis J, Islam R, O’Connor D, Patey A, Ivers N (2017). A guide to using the theoretical domains Framework of behaviour change to investigate implementation problems. Implement Sci.

[31] Gellman MD, Turner JR (2013). Encyclopedia of behavioral medicine.

[32] Glasgow RE, Vogt TM, Boles SM (1999). Evaluating the public health impact of health promotion interventions: the RE-AIM framework. Am J Public Health.

[33] Reilly KL, Nathan N, Wiggers J, Yoong SL, Wolfenden L (2018). Scale up of a multi-strategic intervention to increase implementation of a school healthy canteen policy: findings of an intervention trial. BMC Public Health.

[34] Olstad DL, Lieffers JR, Raine KD, McCargar LJ (2011). Implementing the Alberta nutrition guidelines for children and youth: in a recreational facility. Can J Diet Pract Res.

[35] Prochaska JO, Redding CA, Evers KE. The transtheoretical model and stages of change. Health behavior: Theory, research, and practice. 2015;97.

[36] Seward K, Wolfenden L, Finch M, Wiggers J, Wyse R, Jones J (2017). Improving the implementation of nutrition guidelines in childcare centres improves child dietary intake: findings of a randomised trial of an implementation intervention. Public Health Nutr.

[37] Wolfenden L, Nathan N, Janssen LM, Wiggers J, Reilly K, Delaney T (2017). Multi-strategic intervention to enhance implementation of healthy canteen policy: a randomised controlled trial. Implement Sci.

[38] Yoong SL, Nathan N, Wolfenden L, Wiggers J, Reilly K, Oldmeadow C (2016). CAFÉ: a multicomponent audit and feedback intervention to improve implementation of healthy food policy in primary school canteens: a randomised controlled trial. Int J Behav Nutr Phys Act.

[39] Australian Bureau of Statistics. Australian Standard Geographical Classification (ASGC) 2011 [Available from: https://www.abs.gov.au/websitedbs/D3310114.nsf/home/Australian+Standard+Geographical+Classification+(ASGC)#:~:text=The%20Australian%20Standard%20Geographical%20Classification%20%28ASGC%29%20was%20used,2011%20edition%20of%20the%20ASGC%20was%20the%20last.

[40] Tabak RG, Khoong EC, Chambers DA, Brownson RC (2012). Bridging research and practice: models for dissemination and implementation research. Am J Prev Med.

[41] Keogh-Brown M, Bachmann M, Shepstone L, Hewitt C, Howe A, Ramsay CR et al. Contamination in trials of educational interventions. 2007. 10.3310/hta11430 17935683

[42] Lambert J. Where it all started: The center for digital storytelling in California. Story circle: Digital storytelling around the world. 2009:77–90.

[43] Smeda N, Dakich E, Sharda N (2014). The effectiveness of digital storytelling in the classrooms: a comprehensive study. Smart Learn Environ.

