# Supplementary material for: The impact of an e-newsletter or animated video to disseminate outdoor free-play information in relation to COVID-19 guidelines in New South Wales early childhood education and care services: a randomised controlled trial
Source: BMC Public Health. 2023 Jul 7;23:1306. doi: 10.1186/s12889-023-16177-7 (PMC10326923; doi:10.1186/s12889-023-16177-7)
Supplement: Supplementary file 1 — Supplementary Material 1 [file 12889_2023_16177_MOESM1_ESM.docx]

**Additional File 1: Control Message**

| We are writing to ensure you are aware of the NSW Department of Education COVID-19 Guidelines for ECEC services which can be found [here](https://education.nsw.gov.au/early-childhood-education/coronavirus/advice-for-services-and-providers). The Guidelines include a wide range of topics to assist with preparing for, preventing and managing an outbreak of COVID-19.  While it’s not always possible in an ECEC service to maintain physical distancing, increasing outdoor play and altering meal times can help minimise close contract.  Specifically, the COVID-19 Guidelines recommend that:   - Whenever possible (weather dependent) and when you have enough staffing for adequate supervision, consider operating an indoor/outdoor program for the full day/session. This naturally provides for more space for the children and the setup of more activities for children to engage in. - If you are not able to run an indoor/outdoor program, consider spending more time outdoors, consider the placement of activities and the amount of activities in the outdoor space. A greater range of activities will encourage children to spread out more broadly. - Sharing of food should be actively monitored and discouraged. - Staff should serve children and avoid allowing children to self-serve from a shared plate. - Look at your setup when children are eating. Consider having less children at each table and use more tables to allow more space between children. - If you have limited tables and normally have all children eating at the same time, consider staggered timings of snacks and lunch over a longer period of time.     We hope that you find this information useful and hope that all your staff and children are keeping safe and well.  Kind regards,  *The Good for Kids Team* |
| --- |
