# Supplementary material for: The impact of an e-newsletter or animated video to disseminate outdoor free-play information in relation to COVID-19 guidelines in New South Wales early childhood education and care services: a randomised controlled trial
Source: BMC Public Health. 2023 Jul 7;23:1306. doi: 10.1186/s12889-023-16177-7 (PMC10326923; doi:10.1186/s12889-023-16177-7)
Supplement: Supplementary file 2 — Supplementary Material 2 [file 12889_2023_16177_MOESM2_ESM.docx]

**Additional file 2: Barriers to implementing the Guidelines survey items**

| **TDF construct** | **Survey item** |
| --- | --- |
| Beliefs about consequences | I believe offering an indoor-outdoor program for the full day or offering more time outdoors according to the Guidelines, will benefit public health. |
|  | I believe offering an indoor-outdoor program for the full day or offering more time outdoors according to the Guidelines, will lead to benefits for the children. |
|  | In my view, offering an indoor-outdoor program for the full day or offering more time outdoors according to the Guidelines, is practical. |
| Beliefs about capabilities | I am confident that I can offer an indoor-outdoor program for the full day and offer more time outdoors according to the Guidelines. |
|  | I am capable of offering an indoor-outdoor program for the full day and offer more time outdoors according to the Guidelines, even when little time is available. |
|  | For me, offering an indoor-outdoor program for the full day and more time outdoors according to the Guidelines, is easy. |
| Social influences | People who are important to me think that I should offer an indoor-outdoor program for the full day and offer more time outdoors according to the Guidelines. |
|  | People whose opinion I value approve of me offering an indoor-outdoor program for the full day and more time outdoors according to the Guidelines. |
|  | I can count on support from colleagues whom I work with when things get tough to offer an indoor-outdoor program for the full day and offer more time outdoors according to the Guidelines. |
